# Changes in hemodynamic response function components reveal specific changes in neurovascular coupling in type 2 diabetes

**DOI:** 10.3389/fphys.2022.1101470

**Published:** 2023-01-10

**Authors:** João Valente Duarte, Catarina Guerra, Carolina Moreno, Leonor Gomes, Miguel Castelo-Branco

**Affiliations:** ^1^ Coimbra Institute for Biomedical Imaging and Translational Research (CIBIT), Institute for Nuclear Sciences Applied to Health (ICNAS), University of Coimbra, Coimbra, Portugal; ^2^ Faculty of Medicine, University of Coimbra, Coimbra, Portugal; ^3^ Intelligent Systems Associate Laboratory (LASI), Coimbra, Portugal; ^4^ Service of Endocrinology, Diabetes and Metabolism, Coimbra University Hospital, Coimbra, Portugal

**Keywords:** brain imaging, cerebral hemodynamics, diabetes, fMRI, BOLD signal, neurovascular coupling, hemodynamic response function

## Abstract

Type 2 Diabetes Mellitus (T2DM) is a metabolic disease that leads to multiple vascular complications with concomitant changes in human neurophysiology, which may lead to long-term cognitive impairment, and dementia. Early impairments of neurovascular coupling can be studied using event-related functional magnetic resonance imaging (fMRI) designs. Here, we aimed to characterize the changes in the hemodynamic response function (HRF) in T2DM to probe components from the initial dip to late undershoot. We investigated whether the HRF morphology is altered throughout the brain in T2DM, by extracting several parameters of the fMRI response profiles in 141 participants (64 patients with T2DM and 77 healthy controls) performing a visual motion discrimination task. Overall, the patients revealed significantly different HRFs, which extended to all brain regions, suggesting that this is a general phenomenon. The HRF in T2DM was found to be more sluggish, with a higher peak latency and lower peak amplitude, relative slope to peak, and area under the curve. It also showed a pronounced initial dip, suggesting that the initial avidity for oxygen is not compensated for, and an absent or less prominent but longer undershoot. Most HRF parameters showed a higher dispersion and variability in T2DM. In sum, we provide a definite demonstration of an impaired hemodynamic response function in the early stages of T2DM, following a previous suggestion of impaired neurovascular coupling. The quantitative demonstration of a significantly altered HRF morphology in separate response phases suggests an alteration of distinct physiological mechanisms related to neurovascular coupling, which should be considered in the future to potentially halt the deterioration of the brain function in T2DM.

## Introduction

Type 2 Diabetes Mellitus (T2DM) is a multifactorial metabolic disorder, representing the sixth leading cause of disability, with an ever-increasing incidence (Stumvoll et al., 2005; McCarthy, 2010; Chatterjee et al., 2017; Yuan and Wang, 2017). T2DM causes macro and microvascular alterations (McCarthy, 2010) which cause long-term damage, dysfunction, and deterioration in several tissues and organs, such as the brain, causing cerebrovascular disease and changes in cerebral hemodynamics (McCarthy, 2010; American Diabetes Association, 2012; American Diabetes Association, 2017; Chatterjee et al., 2017; Yuan and Wang, 2017). Due to its association with an increased risk for neural functional loss, long-term cognitive impairment, and dementia (Biessels and Reijmer, 2014; Ryan et al., 2014; Duarte et al., 2015; Moran et al., 2015; Callisaya et al., 2018; Moran et al., 2019; Biessels et al., 2020), it becomes crucial to understand the neurobiological correlates of early brain dysfunction in T2DM, in particular, at early stages of the disease when there are no evident structural lesions (Espeland et al., 2013; Moran et al., 2013; Biessels and Reijmer, 2014).

Several authors have used functional magnetic resonance imaging (fMRI) blood oxygenation level-dependent (BOLD) signal (Ogawa et al., 1990a; Ogawa et al., 1990b) to indirectly measure neuronal activity and investigate neurophysiological impairment in T2DM. However, results in the clinical setting have been inconsistent, namely regarding the presence or absence of functional alterations and its association with cognition (Zhou et al., 2010; Chhatwal and Sperling, 2012; Musen et al., 2012; Xia et al., 2013; Cui et al., 2014; Wang et al., 2014; Liu et al., 2018, 2019; Xiong et al., 2020). The BOLD signal is an intrinsic hemodynamic signal which depends on the neurovascular coupling (NVC) between neuronal activity and cerebral blood flow (Attwell et al., 2010; Phillips et al., 2015). On the other hand, neurovascular coupling reflects the close temporal and regional linkage between neural activity and cerebral blood flow. This close matching of local blood flow to neuronal activation is related to the high energetic demands of brain cells. Thus, even slight changes to the energy supply require precise autoregulation mechanisms. Therefore, an impaired NVC can result in brain dysfunction and neuronal atrophy. Abnormal NVC has been implicated in Alzheimer’s disease, multiple sclerosis, traumatic brain injury, spinal cord injury, and stroke (Fletcher et al., 2022).

Notably, in diabetes, pathophysiological vascular changes can influence the blood flow regulation in cerebral microvasculature, possibly impairing the NVC. Therefore, the interaction of neurons with the surrounding vasculature in terms of autoregulatory mechanisms is critical for proper function of the central nervous system. Their deregulation may contribute to early cognitive impairments, namely in the memory domain. Indeed, several longitudinal studies have shown that patients with T2DM have an increased risk of developing dementia (Hardigan et al., 2016). However, alterations in the BOLD signal may reflect abnormal neuronal activity or inefficient neurovascular coupling, generating an ambiguity in interpretation. Thus, underlying mechanistic changes might be indistinguishable. They could be neurodegenerative, vascular, or a combination of both.

The vascular response triggered by neuronal activation and measured with fMRI BOLD is described by a hemodynamic response function (HRF) (Carusone et al., 2002), which has been shown to be altered, for instance, in patients with stroke (Bonakdarpour et al., 2007; Altamura et al., 2009), resulting in misinterpretation or under-estimation of the fMRI signal. In a previous fMRI study (Duarte et al., 2015), we have used a performance-matched visual stimulation task, thereby recruiting similar neural and cognitive resources in T2DM and control participants, to examine the BOLD response specifically in three task-related regions-of-interest (ROIs) involved in visual processing, perceptual decision mechanisms, and executive functioning (Duarte et al., 2017). This earlier study suggested an overal change in HRF at early stages of T2DM, which might reflect an impaired neurovascular coupling (Duarte et al., 2015), raising the question of which components of the HRF might be changed in diabetes.

This previous study highlighted the need to obtain a complete understanding of the components of hemodynamic model parameters and the underlying physiology of the BOLD signal to improve the utility of functional brain mapping in the context of diabetes.

In this new study, we investigated whether the HRF in its distinct phases would be compromised in individuals with T2DM depending on the brain region or would instead represent a general cortical phenomenon, in a large cohort of patients with T2DM and healthy controls, in response to the same visual motion discrimination task presented with both block and event-related paradigms. The analysis of the data measured during block stimulation allowed to localize activated brain regions, in which we extracted the HRF during the event-related stimulation task by a deconvolution GLM analysis. Overall, and as expected, patients with T2DM revealed significantly different HRF profiles. Furthermore, as a novel approach, several HRF parameters corresponding to distinct phases were extracted from response profiles and compared between groups in each region to understand what sort of physiological changes arise with T2DM and what consequences they can cause.

Since alterations in the HRF may reflect changes in the NVC and, consequently, pathophysiological brain changes even at early stages, the use of the HRF as a functional imaging biomarker has been investigated in the context of cerebral pathology (Miezin et al., 2000; Taylor et al., 2018). While previous studies investigating HRF parameters as biomarkers have focused on peak amplitude (Peck et al., 2004), there has been interest in the evaluation of other parameters, such as the activation duration and the peak latency (Miezin et al., 2000; Bonakdarpour et al., 2007; Lindquist and Wager, 2007). This is important to sort out, prior to the investigation of HRF as a potential biomarker of clinical/cognitive complications in T2DM. As T2DM induces a decrease in cerebral blood flow (Kelly-Cobbs et al., 2012), reducing neural efficiency (Abdelkarim et al., 2019), we hypothesized that T2DM leads to neurovascular decoupling (Duarte et al., 2015) and yields consequences in the HRF morphology components (Hillman, 2014). We hypothesize that T2DM is characterized by a lower overall amplitude of the peak, initial dip and undershoot, and a higher peak latency and that the source of these differences may be possibly driven by the disruption of the NVC.

## Materials and methods

### Participants

We included 141 participants in this study, divided in a group of 64 patients with T2DM and a group of 77 healthy controls. The participants were recruited and included in this analysis according to the procedure described in our previous study (Duarte et al., 2015). The prior study included 51 patients with T2DM and 29 healthy controls, which were also included in the current study with an extended sample size for a detailed analysis of the hemodynamic response function. While in our previous studies (Duarte et al., 2015; Ferreira et al., 2017), the strategy was to have carefully matched cohorts, in the current study, we widely extended the sample size with a focus on using covariates in statistical analysis. All participants provided informed written consent. The Helsinki Declaration of 1975 (and as revised in 1983) guidelines were followed throughout the study. The Ethics Committee of the Faculty of Medicine of the University of Coimbra approved all experimental procedures. Table 1 details the participants’ demographic and clinical data.

**TABLE 1:** Characteristics of the study participants and identification of covariates.

	T2DM	Controls	*p*-value
*n*	64	77	
Age (years)	58.8 ± 8.9	51.4 ± 8.7	<0.001
Gender (M/F)	42/22	32/45	0.004
HbA1c (NGSP, %)	9.55 ± 2.35	5.37 ± 0.36	<0.001
Discrimination threshold (deg/sec)	2.53 ± 1.87	1.39 ± 1.25	<0.001

Data are mean ± standard deviation. NGSP, national glycohemoglobin standardization program; M, male; F, female.

### Experimental protocol

**FIGURE 1:**
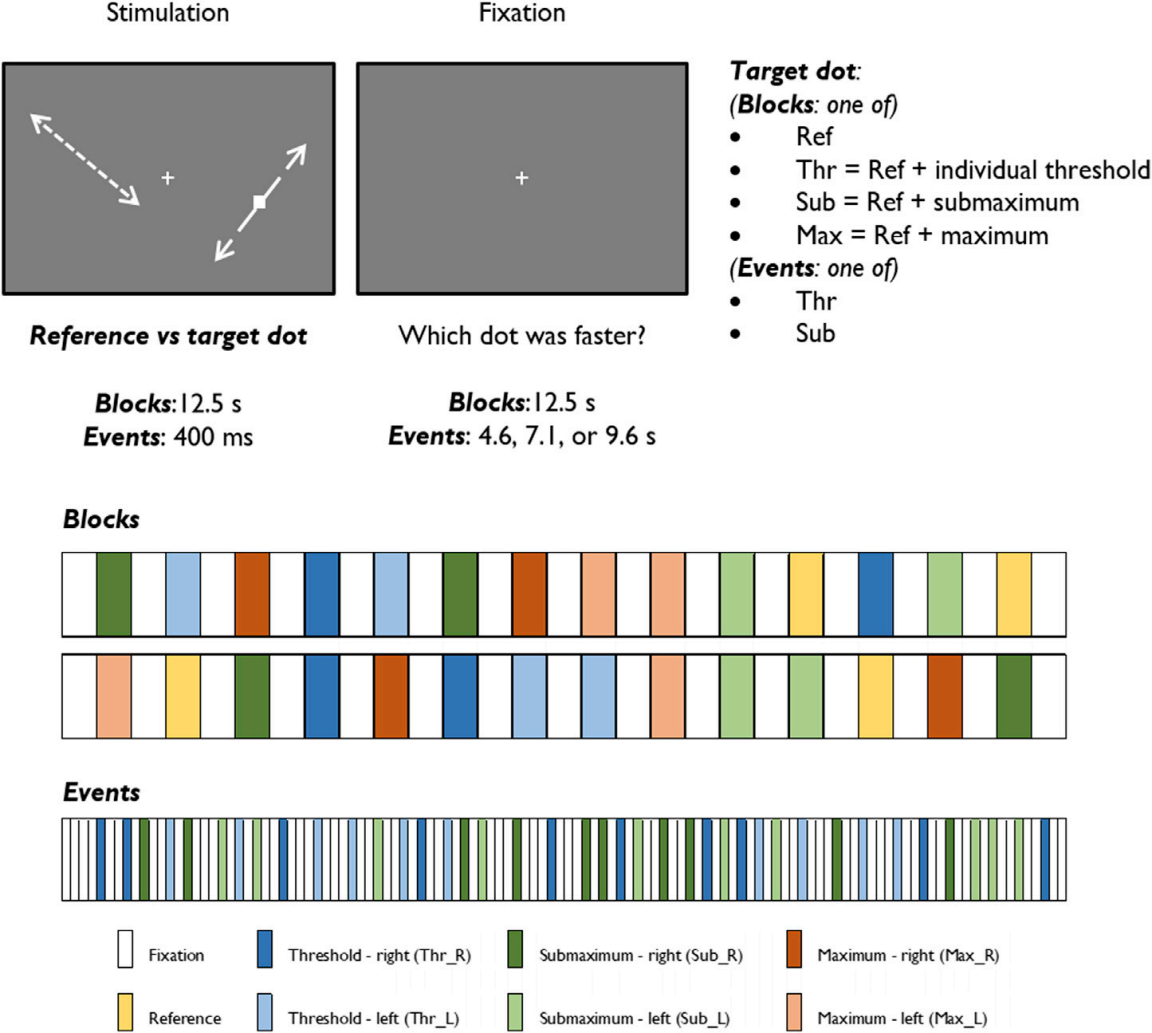
Graphical representation of the stimuli and experimental protocols of block and event-related designs.

We employed both a block and an event-related experimental design to investigate the BOLD response to a visual speed discrimination task, as described in our previous study (Duarte et al., 2015). Briefly, the participants performed a psychophysical task inside the scanner to select stimulus levels individually, which consisted of a two-alternative forced choice test aimed to determine a speed discrimination threshold by comparing the speed of two white dots, the reference and the target. The computation of the speed values was individually tuned to ensure that we would analyze fMRI signal changes in identical performance conditions across participants. Then, each participant performed three fMRI experimental runs: two presenting the task in blocks and one in an event-related design. In the block design, the reference dot constantly moved at 5 deg/s, and the target dot moved with one of four different values: the reference speed (reference condition, most difficult), the reference speed incremented with the individual unit threshold of discrimination (threshold condition, second most difficult), the reference speed incremented with three times the previous threshold (submaximum condition, second easiest), and an arbitrarily defined high-speed value of 20 deg/s (maximum condition, the easiest). The reference condition was presented two times, in which the dot moved at the reference speed in both visual hemifields. Each of the three remaining conditions was presented four times, with the faster dot appearing two times in each visual hemifield. This yields 29 blocks of alternated visual stimulation (14 blocks of 12.5 s each) and baseline fixation (15 blocks of 12.5 s each). In the event-related stimulation paradigm, the alternation between stimulation and baseline fixation is maintained, but only the threshold and the sub-maximum conditions were presented, for the sake of time. Each of the two conditions, representing intermediate difficulty levels, was presented 20 times (10 times per hemi-field). Each visual stimulation period lasted 400 ms, and the baseline fixation period was jittered and lasted 4.600, 7.100, or 9.600 ms, which occurred randomly. The participants were instructed to maintain fixation on a white cross during the whole experiment and report the faster dot during the baseline fixation periods succeeding the stimulation blocks/events. All participants were presented with the same randomized sequences. Both block and event-related designs are represented in Figure 1.

### MRI data acquisition

Functional and structural MRI data were collected at ICNAS, University of Coimbra. The data were acquired on a 3T MR scanner (Magnetom TIM Trio, Siemens, Munich, Germany) with a phased array 12-channel head coil. We acquired a 3D anatomical MPRAGE scan (TR = 2,530 ms; TE = 3.42 ms; FA = 7°; 176 slices with voxel size 1 × 1 × 1 mm) and three functional imaging series consisting of two runs of 145 GE-EPI brain scans (TR = 2,500 ms; TE = 30 ms; FA = 90°; 36 interleaved slices with voxel size 3 × 3 × 3 mm) in a block design stimulation paradigm and one run of 116 GE-EPI scans (with the same parameters) in an event-related design stimulation paradigm.

### fMRI data preprocessing

Structural and functional MRI data were processed as in our previous study (Duarte et al., 2015), using BrainVoyager 21.4 (Brain Innovation, Maastricht, Netherlands). In brief, structural scans underwent skull stripping and intensity inhomogeneity correction. In functional data, we applied slice timing correction, linear trend removal, motion correction, slight spatial smoothing (full width at half maximum of 3 mm), and temporal high-pass filtering (0.02 Hz for block design and 0.04 Hz for event-related design). Functional scans were coregistered with each individual anatomical scan and normalized into MNI space.

### fMRI data analysis

Statistical analysis of fMRI data was performed using the RFX GLM framework, which allows to explicitly model both within-subjects and between-subjects variance components to generalize findings at the population level (Beckmann et al., 2003; Penny and Holmes, 2003).

#### Block design–standard GLM

Predictors for each stimulation condition were used to estimate condition effects (beta weights) separately for each subject. In the second step, the beta weights of all participants were provided as an input for a second-level group analysis, in which group effects of all conditions were estimated as a percent signal change relative to the baseline. To locate the ROIs with overall engagement in the stimulation task, a [*stimulation* vs. *baseline*] contrast was defined including all stimulation conditions. The resulting statistical map, presented in Figure 3, was corrected for multiple comparisons with the Bonferroni correction for a significance threshold of *p* < 0.05. The clusters of voxels showing significant signal changes were defined as ROIs to be further investigated in the subsequent analysis of the HRF parameters, which were calculated after estimation of the HRF with deconvolution GLM analysis of the event-related run. The ROIs were differentiated into positive and negative signal change ROIs, and their list, including the MNI coordinates of each cluster, is presented in Table 2.

**TABLE 2:** Clusters with positive signal change during stimulation blocks.

Anatomical brain region	Cluster code	MNI coordinates at the cluster’s peak				
		X	Y		Z	
Left Inferior Parietal Lobule, Brodmann area 40	L_IPL_BA40	−30	−46		40	
Left Insula, Brodmann area 13	L_Insula_BA13	−30	23		4	
Left Precuneus, Brodmann area 7	L_Precuneus_BA7	−24	−52		44	
Right Inferior Frontal Gyrus, Brodmann area 9	R_IFG_BA9	45	11		25	
Right Middle Frontal Gyrus, Brodmann area 8	R_MFG_BA8	6	17		46	
Right Middle Frontal Gyrus, Brodmann area 46	R_MFG_BA46	48	26		25	
Right Superior Frontal Gyrus, Brodmann area 6	R_SFG_BA6	6	8		53	
Right Superior Parietal Lobule, Brodmann area 7	R_SPL_BA7	30	−57		46	
Right V2 area, Brodmann area 18	R_V2_BA18	29	−73		22	
Right V5/MT area, Brodmann area 19	R_MT_BA19	46	−64		9	

#### Event-related design–deconvolution GLM

For the event-related design, we applied a deconvolution GLM analysis within each previously defined ROI to separate the contributions of different events and estimate the response curves (HRF) for each condition (Buckner, 1998; Glover, 1999). Deconvolution GLM is an alternative GLM analysis in which the shape of the HRF is not fixed in advance. Each protocol condition encodes a set of stick predictors, each of them separately estimating the HRF amplitude at a data point regarding the onset of that condition. In the end, the series of amplitude estimates describes the HRF shape in each condition.

### HRF analysis

After the GLM deconvolution analysis, the series of beta weights that describe the estimated HRF in each stimulation condition, for each subject, and in each ROI, were analyzed in MATLAB.

#### Average and median HRF curves

The mean beta weights for each condition (Threshold and Submaximum) were calculated for each ROI by averaging the corresponding left and right beta values for each participant. Then, for each group, condition, and ROI, the average and standard deviation of each beta weight were calculated, yielding an average HRF. We also calculated the median HRF with its interquartile range, as it is a more robust measure and gives an enhanced sense of a typical value, with less influence from outliers. Finally, we also calculated the average and median HRFs per ROI type (positive or negative signal change) in each group and stimulation condition, which were designated as grand average and grand median HRFs, and are depicted in Figures 4, 6.

#### Estimation of the HRF parameters

**FIGURE 2:**
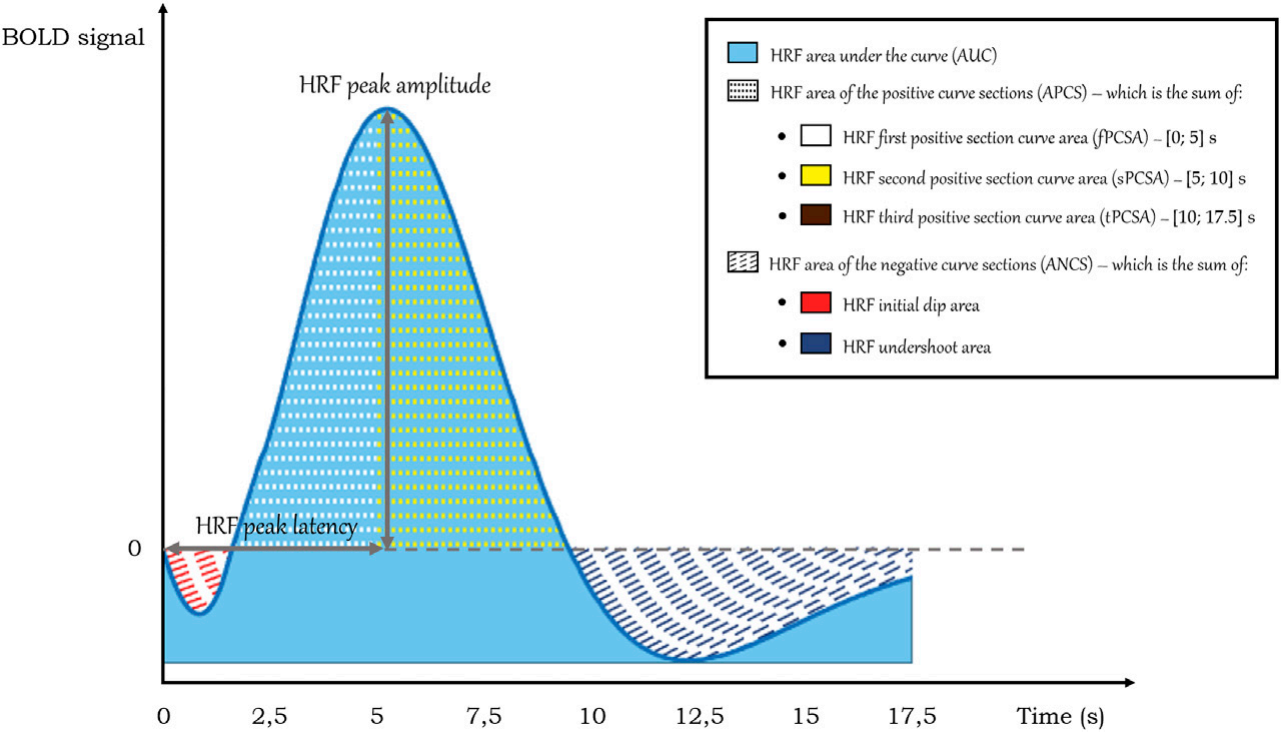
HRF parameters calculated on the HRF curves. Note that the HRF third positive section curve area is not depicted in this representation of a standard HRF but was observed in cases of altered HRF in T2DM.

Several parameters describing the HRF morphology were computed and compared between groups per ROI to assess whether there were any differences and, if so, if they could provide any clue regarding underlying neurovascular damage (Bellgowan et al., 2003). The HRF parameters that were calculated are portrayed in Figure 2.

The calculated HRF parameters included peak amplitude, peak latency, relative slope to peak, area under the curve (AUC), area of the positive curve sections (APCS) and area of the negative curve sections (ANCS). In healthy participants, the canonical HRF peaks on average at about 6–8 s after a short dip and then has an undershoot that lasts for as long as 15 s post-onset before returning to baseline (Bonakdarpour et al., 2015). As it can vary in this clinical context, we calculated the HRF peak amplitude by determining the HRF curve maximum in the range of data points between 5 and 15 s. In this estimation, it was also determined the data point where the peak occurs, which, when multiplied by the repetition time, yields the HRF peak latency. In turn, the HRF relative slope to peak was given by the ratio between the HRF amplitude variation between the peak time and the initial instant (*t* = 0) and the peak latency. The HRF area under the curve (AUC) was determined by applying the trapezoidal rule between the onset and the end of the HRF after offsetting its values to set its minimum value as the zero of the HRF. Regarding the HRF area of the positive curve sections (APCS), the trapezoidal rule was implemented between the positive HRF values and zero. Likewise, for the HRF area of the negative curve sections (ANCS), the same approach was employed, but for the negative HRF values.

As the HRF is observed to show considerable variation in T2DM, we also calculated separate parts of the positive and negative curve sections to account for differences between groups in initial dip, undershoot and sustained or quickly dropping responses after the peak. We calculated the initial dip area, the undershoot area, the first positive section curve area [fPCSA, in the interval (0; 5) s], the second positive section curve area [sPCSA, in the interval (5; 10) s], and the third positive section curve area [tPSCA, in the interval (10; 17.5) s].

#### Coefficient of variation

To ascertain how reliable and/or robust the mean peak and peak latency were, we calculated their coefficient of variation (CV), which represents the variability of these parameters concerning the population’s average, and it is given by the ratio between the standard deviation of the parameter and its corresponding mean value.

### Statistical analysis of the HRF parameters

The parameters of the average HRF were calculated for each participant, in each ROI, for each stimulation condition. As the values were very similar across ROIs, the average parameters were calculated for all positive and negative ROIs in each participant for comparison between groups. The differences between T2DM and CNT were assessed with an ANCOVA model, in which the HRF parameters were the dependent variable and the group was the between-subjects factor, while accounting for the effect of age as a covariate. The statistical tests were corrected for multiple comparisons with the Benjamini—Hochberg approach, and the *p*-values were adjusted for false discovery rate (FDR). We further calculated the correlation of each HRF parameter with age and HbA1c levels in each group, as well as the difference between parameters estimated in participants of each sex.

## Results

### Standard GLM analysis of the block design

**FIGURE 3:**
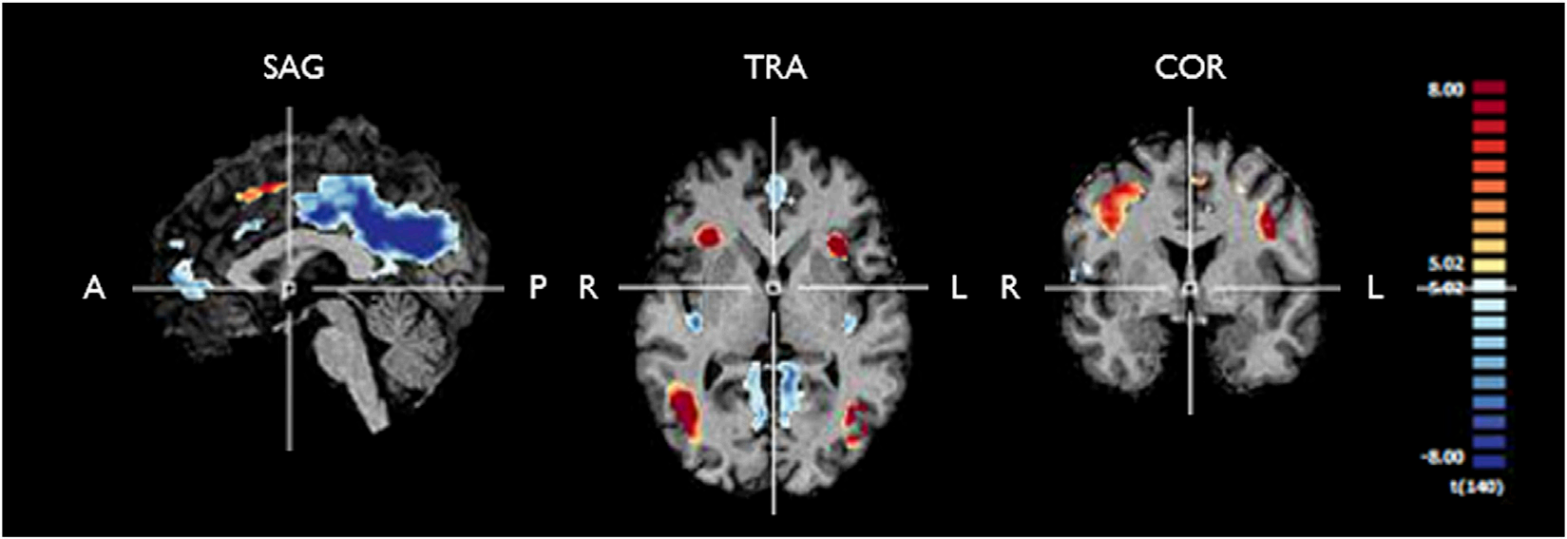
Functional maps generated from RFX GLM analysis of the fMRI response to any stimulation condition during the block design experiment for controls and T2DM patients. The map is corrected for multiple comparisons (p_Bonferroni_ < 0.05). One can see significant positive signal change (yellow/red) and negative signal change (blue) in several clusters, which are further described in Tables 2, 3. We used these ROIs to extract the fMRI BOLD signal for further analysis of the HRF in each group. SAG, sagittal; TRA, transverse; COR, coronal; A, anterior; P, posterior; R, right; L, left.

The fMRI statistical map extracted from the standard RFX GLM analysis of the block design task is shown in Figure 3. In the blocked experiment, we can observe similar statistical maps as those obtained in our previous study, including the visual motion regions, the insula, and the inferior frontal gyrus (Duarte et al., 2015). The list of regions showing significant positive and negative signal change is presented in Tables 2, 3, respectively.

**TABLE 3:** Clusters with negative signal change during stimulation blocks.

Anatomical brain region	Cluster code	MNI coordinates at the cluster’s peak				
		X	Y		Z	
Left Anterior Cingulate, Brodmann area 32	L_AC_BA32	0	44		1	
Left Cingulate Gyrus, Brodmann area 31	L_CG_BA31	−3	−46		32	
Right Cingulate Gyrus, Brodmann area 24	R_CG_BA24	−3	−10		43	
Right Insula, Brodmann area 13	R_Insula_BA13	37	−19		4	
Left Posterior Cingulate, Brodmann area 30	L_PC_BA30	−6	−55		13	
Right Posterior Cingulate, Brodmann area 23	R_PC_BA23	−3	−45		28	
Left Paracentral Lobule, Brodmann area 5	L_PrcL_BA5	−12	−37		49	
Left Parahippocampal Gyrus, Brodmann area 36	L_PrhG_BA36	−30	−31		−17	
Right Precentral Gyrus, Brodmann area 4	R_PrecG_BA4	36	−19		40	
Right Precentral Gyrus, Brodmann area 43	R_PrecG_BA43	54	−7		11	
Right Primary Sensorial area, Brodmann area 1	R_PrimSens_BA1	39	−16		19	
Right Superior Temporal Gyrus, Brodmann area 39	R_STG_BA39	52	−55		19	

### Average and median HRF

#### ROIs with positive signal change

**FIGURE 4:**
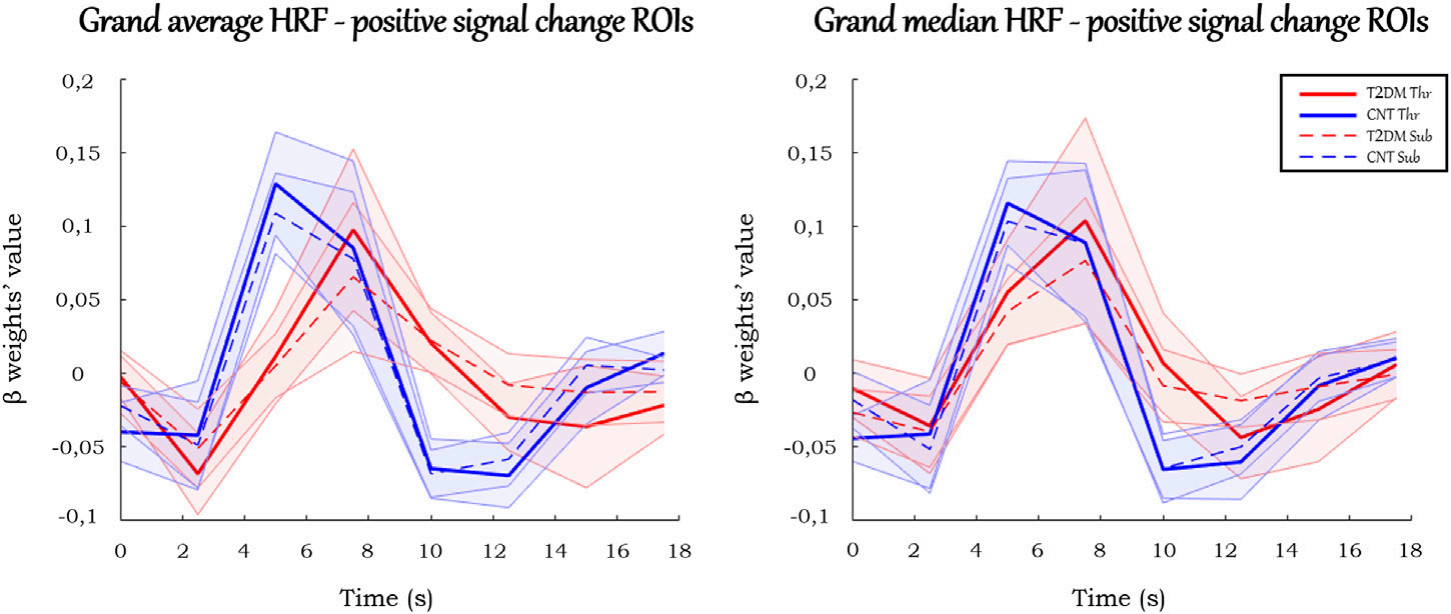
Grand average and grand median HRF curves in the ROIs with positive signal change. Solid and dashed thick lines represent the average/median for each condition, and the shaded areas represent the standard deviation/interquartile range.

**FIGURE 5:**
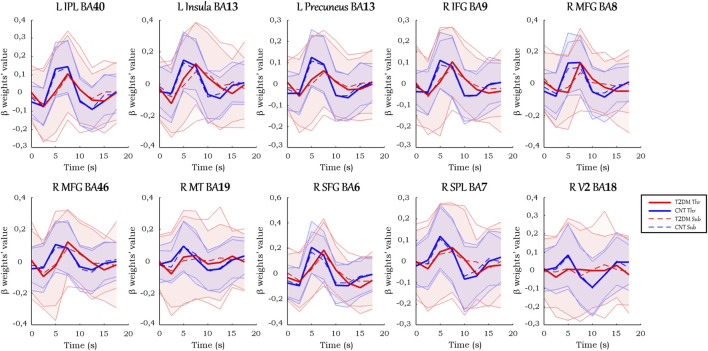
Average HRF curves in each ROI with positive signal change. Solid and dashed thick lines represent the average for each condition, and the shaded areas represent the standard deviation.

In the ROIs with positive signal change, on average (Figure 4), T2DM participants showed a more delayed HRF, with smaller values of peak amplitude, relative slope to peak, AUC, PCSA, and NCSA, and higher peak latency. Furthermore, the HRF of T2DM participants depicted a larger variability, a deeper initial dip, and an absent or lengthier but less intense undershoot with later onset. These results are observed consistently in the average HRF plots in individual ROIs, presented in Figure 5. Similarly, the grand median HRF plot, displayed in Figure 4, reveals an overall replication effect, which shows that these results do not depend on outliers.

#### ROIs with negative signal change

There were ROIs with negative signal change in the block paradigm in which healthy controls showed a positive response whereas T2DM participants showed a negative response, which suggests a possible abnormal enhancement of the dip signal due to an excess of oxygen consumption.

**FIGURE 6:**
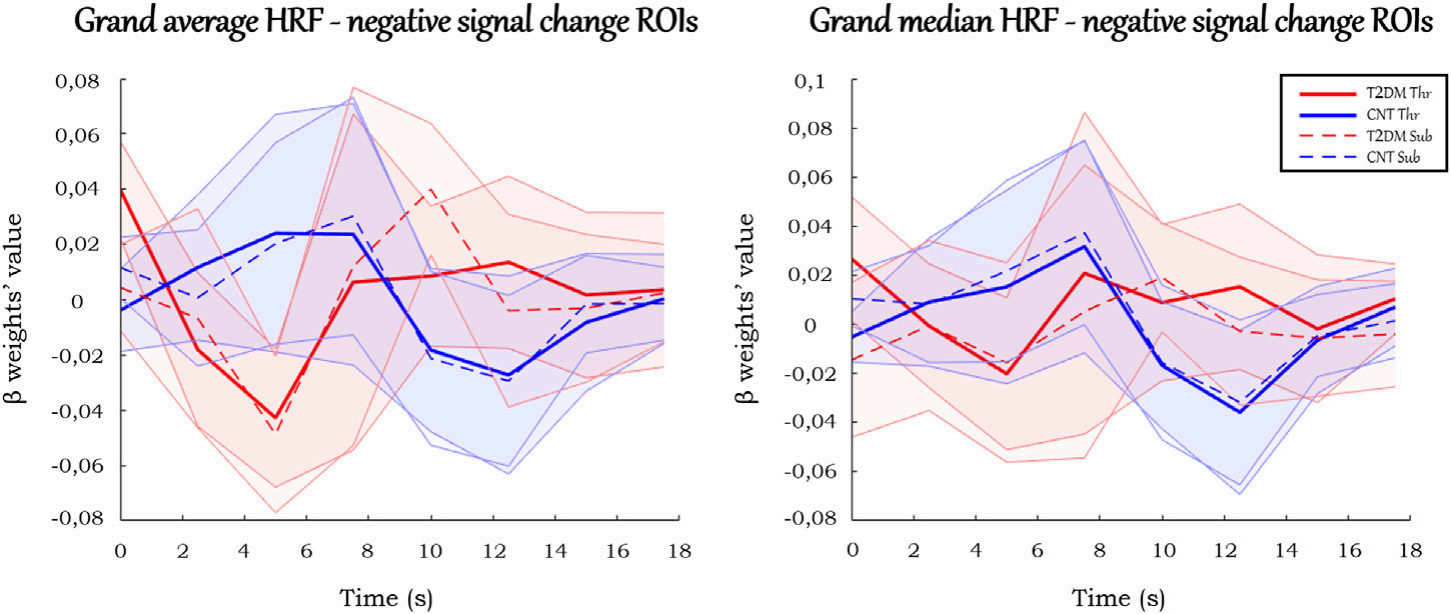
Grand average and grand median HRF curves in the ROIs with negative signal change. Solid and dashed thick lines represent the average/median for each condition, and the shaded areas represent the standard deviation/interquartile range.

The HRFs in these ROIs (Figure 6) displayed less amplitude variation than the analogs in the positive signal change ROIs. Besides, they presented atypical responses, which made rendering estimation for several HRF parameters in these ROIs challenging (see Supplementary Material S1). Their values showed different properties from the ones previously described in the positive signal change ROIs. Given the atypical profiles of the regions, which did not follow the standard HRF, they were not further considered for parameter extraction.

### HRF parameters

In the pool of ROIs with standard positive signal change, the average or median parameters of the HRF confirm the observation that, generally, the peak amplitude, slope to peak, and undershoot area are lower in T2DM. On the other hand, the peak latency and the initial dip seem to be higher in T2DM. The summary of these results, with the statistical comparison between groups, is presented below in Table 4.

**TABLE 4:** Average (±SD) HRF parameters in ROIs with positive signal change.

HRF parameter	Condition	T2DM	CNT	Statistics	Adjusted *p*-value
Peak amplitude	Thresh	0.098 ± 0.055	0.130 ± 0.035	1.592	0.177
	Submax	0.072 ± 0.039	0.113 ± 0.029	2.615	0.034
Peak latency	Thresh	7.500 ± 0.796	5.000 ± 1.054	−3.023	0.007
	Submax	7.500 ± 0.796	5.000 ± 1.054	−3.481	0.002
Relative slope to peak	Thresh	0.013 ± 0.008	0.032 ± 0.011	4.140	0.002
	Submax	0.010 ± 0.006	0.025 ± 0.008	4.720	0.001
AUC	Thresh	1.297 ± 0.450	1.413 ± 0.253	0.707	0.489
	Submax	1.007 ± 0.354	1.340 ± 0.218	2.537	0.037
APCS	Thresh	0.324 ± 0.155	0.492 ± 0.127	2.653	0.033
	Submax	0.254 ± 0.127	0.413 ± 0.090	3.217	0.012
fPCSA	Thresh	0.006 ± 0.006	0.128 ± 0.027	3.742	0.001
	Submax	0.004 ± 0.019	0.097 ± 0.022	3.756	0.001
sPCSA	Thresh	0.287 ± 0.160	0.340 ± 0.153	0.759	0.472
	Submax	0.206 ± 0.138	0.293 ± 0.113	1.551	0.182
tPCSA	Thresh	0.027 ± 0.023	0.004 ± 0.022	−1.323	0.219
	Submax	0.039 ± 0.032	0.013 ± 0.019	−1.935	0.083
ANCS	Thresh	0.364 ± 0.156	0.454 ± 0.130	1.401	0.218
	Submax	0.223 ± 0.101	0.394 ± 0.088	4.033	0.002
Initial dip area	Thresh	0.170 ± 0.077	0.121 ± 0.080	−1.403	0.218
	Submax	0.144 ± 0.073	0.110 ± 0.060	−1.140	0.292
Undershoot area	Thresh	0.194 ± 0.128	0.333 ± 0.067	3.047	0.002
	Submax	0.056 ± 0.061	0.284 ± 0.044	3.364	0.001

AUC, area under the curve; APCS, area of the positive curve section; fPCSA, first positive curve section area; sPCSA, second positive curve section area; tPCSA, third positive curve section area; ANCS, area of the negative curve section; Thresh, Threshold; Submax, Submaximum.

## Discussion

In this study, we found that the HRF in the brain of patients with T2DM is altered relative to healthy controls, as shown by a novel quantitative analysis of the HRF morphology. Overall, in all ROIs, T2DM patients presented a more sluggish (time-delayed) HRF, diverging from the canonical HRF, with a higher peak latency and an absent or less intense but lengthier undershoot (curve below the baseline at the end of the response). Furthermore, the HRF in T2DM patients exhibited a smaller peak amplitude and relative slope to peak. These differences were found using age as a covariate, thus suggesting a specific effect of T2DM independent of healthy aging.

Although the area under the curve, including the positive and negative sections, appears to be relatively similar between the two populations, there is still a trend to be smaller in T2DM patients. Besides, the variability of HRF parameters was, on average, higher in T2DM patients. Lastly, the HRF of T2DM patients often included an initial dip (the classic initial reduction due to the mismatch between oxygen consumption and blood supply) with clear enhancement, unlike what is observed in the control group. This even led to some regional dominantly negative responses in diabetic patients in contrast with controls.

These results are consistent with our hypothesis that the underlying reason for these changes in the HRFs of T2DM patients might be an early compromised neurovascular coupling. Possible explanations for the observed differences include vascular damage (such as endothelial dysfunction), impairment of the vasodilation regulatory mechanisms, or excessive O_2_ consumption. It is not likely that different levels of neuronal activity alterations could have a significant effect on these curves because, as mentioned, the stimuli were individually fitted, so that the task difficulty was similar for all participants. Furthermore, the task performance was identical between groups, revealing a comparable perceptual discrimination ability.

In T2DM patients, there is an imbalance between relative O_2_ consumption and blood supply. In fact, given similar neuronal activation conditions in both groups, with NVC changes in T2DM, the oxygenated blood supply becomes smaller, and, thus the O_2_ decrease induced by neuronal activity will not be so swiftly compensated. This contributes to the overall HRF delay observed. Consequently, the deoxyhemoglobin concentration will further increase initially, hence decreasing the BOLD signal, which explains the large initial dip seen in its average and median HRFs, which in turn was most often absent or less perceptible in the controls.

When the blood supply finally starts to balance the O_2_ consumption, the BOLD signal increases, but more gradually and slower than in healthy conditions, until the peak is reached, which elucidates, in turn, the higher peak latency and smaller peak amplitude and relative slope to peak overall seen in the average and median HRFs of T2DM patients. On the opposite, the controls, with a more efficient NVC, show a higher and faster BOLD signal increase, producing a higher peak amplitude, relative slope to peak, AUC, PCSA, and NCSA, but a lower peak latency, which matches with the overall results. In T2DM patients, after reaching the peak, the return to the baseline, due to the aforementioned reasons, will be delayed and will be slower than in controls, which is mainly in line with the achieved results. On the other hand, the undershoot may not be discerned, which may be due to a masking effect prompted by the lower efficiency of the first phase of the hemodynamic response.

The components of the NVC may present intraindividual, inter-region, and inter-condition variability (Elbau et al., 2018). Its characterization to understand how the hemodynamic response is altered in T2DM, is therefore quite relevant. Our results show that the hemodynamic response, did indeed display a large inter-subject variability in T2DM, which is consistent with the notion of damage induced differences in NVC. Conversely, control participants might have a more efficient NVC, and the hemodynamic response will be more similar between them and will show less variability, also taking into account the relatively restricted age of the sample (40–76 years). Furthermore, the results also demonstrate a robust replication of the HRF effect between ROIs and between the type of stimuli. This consistency suggests an overall effect of T2DM on the HRF which is similar between regions and conditions. To further test the roles of variables such as age, sex, or HbA1c levels as possible causes for this variability, we investigated the correlation of the HRF parameters with these factors in each group. We did not find any significant correlation of HRF with age or HbA1c, nor a differential effect of sex, which suggests that these are not the cause of the variability in this data set, but rather T2DM.

In the ROIs with negative signal change, notably, their particular morphology justifies the reason why we designated these ROIs as *negative signal change* ROIs (may be corresponding and exaggerated negative dip) instead of *deactivation regions*. The HRFs of these ROIs were distinct from the classical deactivation pattern, which expectedly would be like those existing in the positive change ROIs, yet inverted. That is, there would be a large signal decrease against the baseline, a peak (which would correspond to the minimum of the function) around 4–6 s, and a return to the baseline at 16–20 s. Similarly to the canonical HRF, there could also be an initial dip (positive) before the peak (Havlicek et al., 2017). Overall, it could be captured as a deactivation by the GLM. As the term *deactivation* is commonly associated with a physiological meaning - a region which decreases its activity during a condition - and as the meaning of the HRFs of these ROIs is not understood, it was considered wiser to designate them as *negative signal change* ROIs. To maintain the coherence, the remaining ROIs, on which the discussion was focused, were designated as *positive signal change* ROIs, in line with their typical *activation* responses.

A very interesting observation is that the same brain region (Insula BA13) showed very distinct HRFs in the two hemispheres. This intriguing finding will need to be investigated in the future, in line with the hemispheric functional asymmetries that are known to exist in this region (Eckert et al., 2009; Uddin, 2015; Intaitė et al., 2016; Zhang et al., 2019; Sayal et al., 2020).

Finally, as a limitation of the study, we should mention that despite the strong statistical power of these results, besides T2DM, there are several other sources of NVC changes, which, in turn, can introduce variability. For instance, the BOLD signal, which measures indirectly the NVC, is also sensitive to other hemodynamic processes, even from non-pathological sources (e.g., atypical brain physiology) (Rossini et al., 2004; Elbau et al., 2018). Thus, when the NVC is investigated, its interpretation and comparison between groups become more complicated by additional factors (Whittaker et al., 2016). Since the NVC is a rather complex phenomenon whose components change between regions and conditions, conditioning how the coupling takes place (Drew, 2019), further research, including in animal models, should be done on this topic.

## Conclusion

This study provides robust evidence that T2DM patients have an early compromised neurovascular coupling, as shown by the changes in the hemodynamic response function overall in the brain. The novel demonstration of a distinct HRF profile is expressed by a more sluggish response (higher peak latency), with a smaller peak amplitude, a pronounced initial dip, a delayed or absent undershoot, a decreased relative slope to peak, and lower positive and negative areas under the curve. These changes in the HRF morphology may provide novel markers of neurovascular uncoupling caused by T2DM.

Therefore, this study reinforces the role of fMRI as a tool to evaluate brain function and neurovascular coupling in T2DM. Duarte et al. (2015) had previously suggested that BOLD signal differences in diabetic patients could potentially identify NVC disruption, which was shown here. On the other hand, the novel analysis of the HRF parameters may prompt further studies that may involve classification and/or its use as a biomarker which might be related to the pathophysiological evolution and, consequently, play a potentially important role in the assessment of disease progression and effects of therapy.

## Data Availability

The raw data supporting the conclusions of this article will be made available by the authors, without undue reservation.
