# Supplementary figures and images for: Changes in hemodynamic response function components reveal specific changes in neurovascular coupling in type 2 diabetes
Source: Front Physiol. 2023 Jan 10;13:1101470. doi: 10.3389/fphys.2022.1101470 (PMC9872943; doi:10.3389/fphys.2022.1101470)

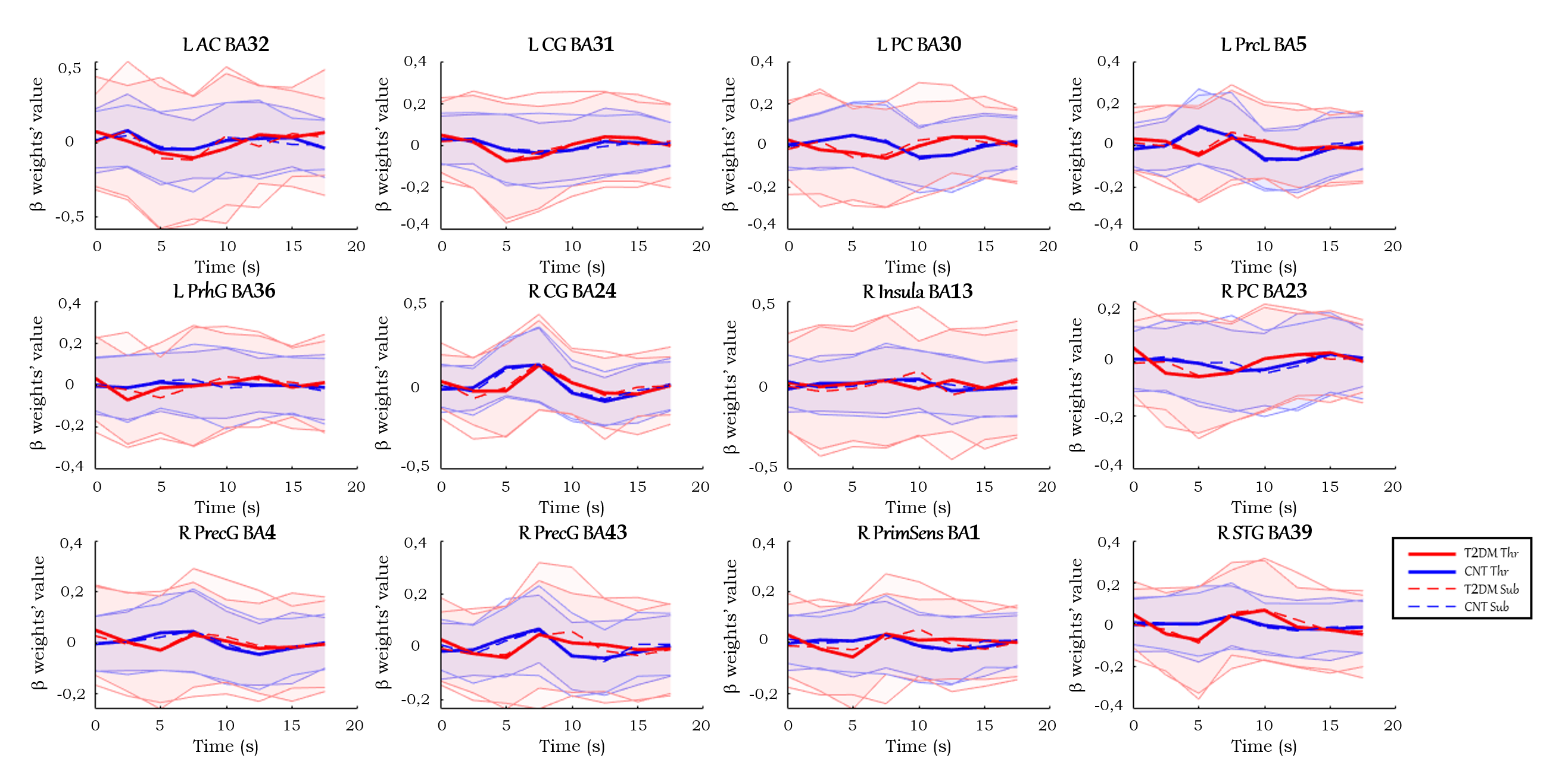

Supplement: Supplementary file 1 [file Image1.tiff]
